# Protein Tyrosine Kinase 7 (PTK7) as a Predictor of Lymph Node Metastases and a Novel Prognostic Biomarker in Patients with Prostate Cancer

**DOI:** 10.3390/ijms150711665

**Published:** 2014-07-01

**Authors:** Hongtuan Zhang, Andi Wang, Shiyong Qi, Shang Cheng, Bing Yao, Yong Xu

**Affiliations:** Department of Urology, National Key Clinical Specialty of Urology, the Second Hospital of Tianjin Medical University, Tianjin Key Institute of Urology, Tianjin 300211, China; E-Mails: urology2003@gmail.com (H.Z.); wangandi123@gmail.com (A.W.); urologydoctorcsy@gmail.com (S.Q.); chengsurology@gmail.com (S.C.); bingyaodoctor1@gmail.com (B.Y.)

**Keywords:** PTK7, prostate cancer, biochemical recurrence-free survival, overall survival

## Abstract

Protein tyrosine kinase 7 (PTK7) has been studied in various tumors, but its role in prostate cancer remains unknown. This study is aimed to investigate the prognostic and predictive significance of PTK7 in patients with prostate cancer. PTK7 expression was evaluated by real-time reverse transcription polymerase chain reaction (RT-PCR) and Western blot analysis in 20 pairs of benign prostatic hyperplasia specimens and prostate cancer specimens. Then, we examined the immunohistochemical expression of PTK7 in 180 prostate cancer specimens and evaluated its clinical significances. Elevated PTK7 expression was significantly associated with lymph node metastases, seminal vesicle invasion, prostate cancer stage, the higher preoperative prostate-specific antigen, the higher Gleason score, angiolymphatic invasion, and biochemical recurrence. The results revealed that the overexpression of PTK7 in prostate cancer was an independent prognostic factor for poor overall survival and biochemical recurrence-free survival. The present data provide evidence that PTK7 predicts lymph node metastasis and poor overall survival and biochemical recurrence-free survival, highlighting its potential function as a therapeutic target for prostate cancer.

## 1. Introduction

Prostate cancer is the second most common malignancy diagnosed in men worldwide, being one of the major causes of cancer-related morbidity and mortality [1]. Prostate cancer-related mortality results from distant metastases [2]. Although most patients with prostate cancer show an indolent clinical course after radical prostatectomy, few experience clinical relapse and die of the disease [3,4]. The first evident sign of relapse following radical prostatectomy is a rising prostate-specific antigen. Prostate-specific antigen is a protein predominantly secreted by prostatic epithelial cells and is one of the best serum biomarkers available [5,6]. A major issue with prostate-specific antigen is its lack of specificity, as other non-malignant diseases of the prostate also display elevated serum prostate-specific antigen levels, which can lead to over-diagnosis [5]. In addition, prostate-specific antigen is a poor indicator of aggressiveness, leading to potential over-treatment of many prostate cancer patients. Therefore, identifying prognostic factors for biochemical recurrence-free survival and overall survival is critical for accurately predicting the prognosis of prostate cancer patients after radical prostatectomy. The prognosis of patients with prostate cancer and lymph node involvement after radical prostatectomy is highly variable [7]. Risk stratification could improve clinical decision-making and personalized post-surgical management [8,9].

The receptor protein tyrosine kinase 7 (PTK7), a pseudokinase, also known as colon carcinoma kinase, was originally identified as a protein overexpressed in colon cancer cell lines [10]. In humans, the full-length membrane PTK7 consists of seven extracellular immunoglobulin-like domains, a transmembrane region, a juxtamembrane region and a catalytically inert cytoplasmic tyrosine kinase domain [11]. Studies showed that PTK7 regulates both the canonical Wnt and the noncanonical Wnt/planar cell polarity pathways [12,13]. In different biological systems, PTK7 interacts, either directly or indirectly, with plexins, semaphorins, the receptor for activated-C kinase 1 and protein kinase C δ1, Wnt3a, Wnt8, and β-catenin [12,13,14,15,16,17,18]. PTK7 has been identified as a protein with an important role not only in embryogenetic tube formation, but also migration and invasion of endothelial and cancer cells *in* *vitro* [19,20]. It has been demonstrated that PTK7 overexpression is observed in several cancers including colon cancer [6], gastric cancer [21], lung cancer [22], acute myeloid leukemia [23], esophageal squamous cell cancer [24], and liposarcoma [25]. Ectopic overexpression of PTK7 in leukemia cells promotes cell migration and survival, whereas knockdown of PTK7 shows the opposite effects [26]. Knockdown of PTK7 in HCT-116 cells also inhibits cell proliferation and induces apoptosis [27]. Similarly, Knockdown of PTK7 has been shown to inhibit proliferation and invasion of liposarcoma cells and induces apoptosis [25].

With regard to the important role of PTK7 in tumorigenesis and its progression, PTK7 overexpression has been studied in several types of tumors tissues and has exhibited clinical relevance. PTK7 plays an important role in the motility and invasivity of cancer cells. The results show that PTK7 may be used as a potential tissue biomarker to avoid overtreatment of non-aggressive prostate cancer [28]. However, the biological significance of PTK7 in human prostate cancer and lymph node involvement has not been investigated so far. We evaluated PTK7 expression in prostate cancer and its prognostic and predictive significances in this study. The data showed the crucial role of PTK7 as a prognostic and lymph node metastasis biomarker as well as a novel potential therapeutic target in prostate cancer.

## 2. Results and Discussion

### 2.1. Results

#### 2.1.1. Expression of Protein Tyrosine Kinase 7 (PTK7) mRNA in Benign Prostatic Hyperplasia and Prostate Cancer Tissues by Quantitative Real Time Reverse Transcriptase Polymerase Chain Reaction (qRT-PCR)

The expression of PTK7 mRNA was detected and analyzed in twenty pairs of prostate cancer and benign prostatic hyperplasia tissues. Compared with benign prostatic hyperplasia tissues, PTK7 expression was 3-fold higher in prostate cancer tissues (*p* = 0.016).

#### 2.1.2. Expression of PTK7 Protein in Benign Prostatic Hyperplasia and Prostate Cancer Tissues by Western Blot Analysis

**Figure 1:**
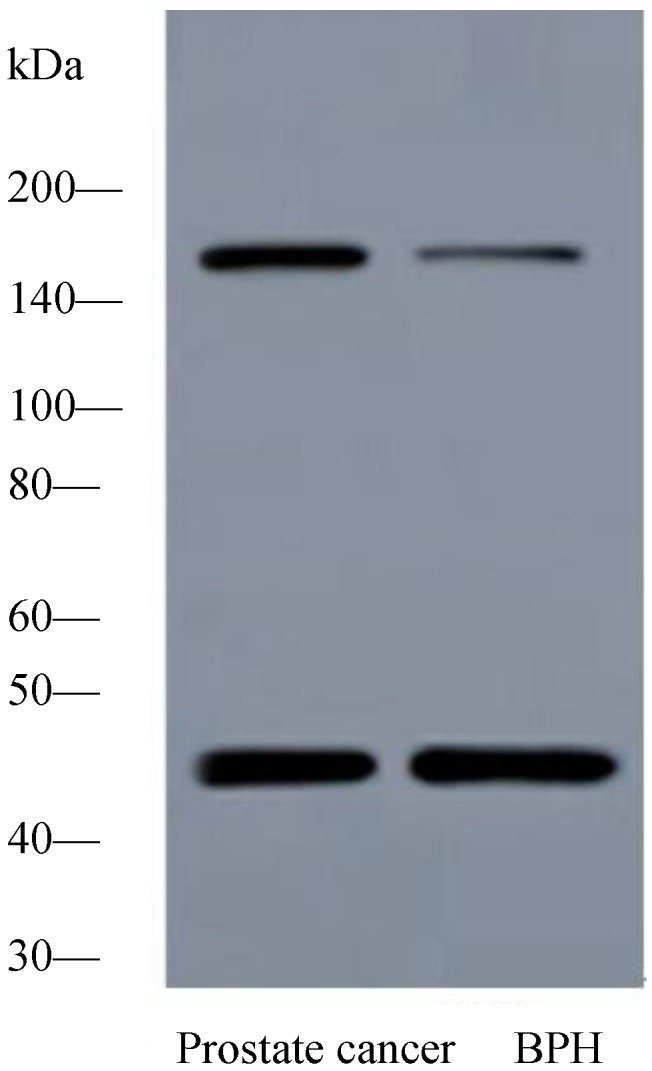
Representative western blot analysis of protein tyrosine kinase 7 (PTK7) expression in prostate tissues. The expression of PTK7 protein was significantly increased in prostate cancer tissues compared with benign prostatic hyperplasia tissues. Protein samples obtained from benign prostatic hyperplasia tissues and prostate cancer tissues were analysed using SDS-PAGE followed by immunoblotting with antibody against PTK7. The levels of β-actin were used as an internal control.

Western blot demonstrated a specific band for PTK7 protein at 118 kDa. The difference in PTK7 expression between prostate cancer and benign prostatic hyperplasia tissues reflected at protein level was investigated using Western blot. Expression of PTK7 protein was significantly elevated in samples of prostate cancer tissues compared with that present in samples of benign prostatic hyperplasia tissues (Figure 1).

#### 2.1.3. Expression of PTK7 Protein in Benign Prostatic Hyperplasia and Prostate Cancer Tissues by Immunothistochemical Staining

**Figure 2:**
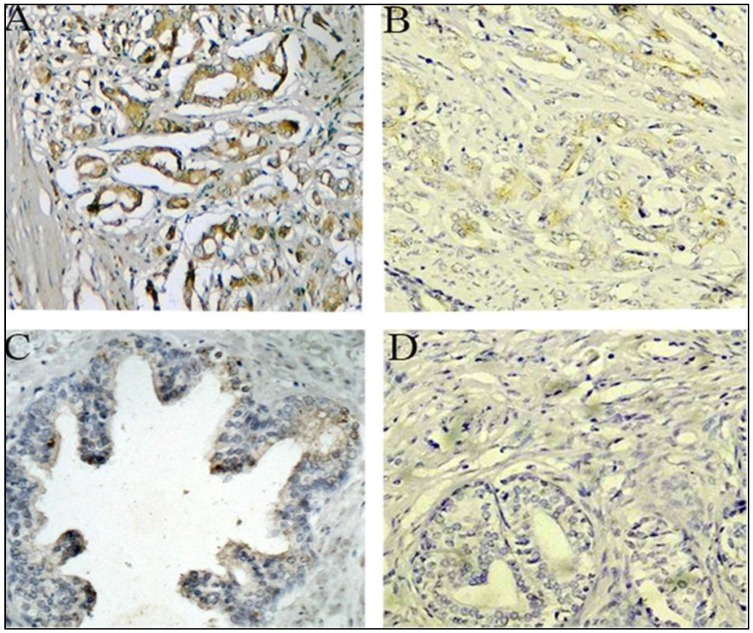
Immunohistochemical staining for PTK7 in prostate cancer and benign prostate tissue (original magnification ×200). (**A**) High PTK7 protein expression was found in cytoplasm of prostate cancer tissues; (**B**) Low PTK7 protein expression was found in cytoplasm of prostate cancer tissues; (**C**) PTK7 weakly positive staining was found in cytoplasm of benign prostate tissue; and (**D**) Negative immunostaining of negative controls with the primary antibody omitted in prostate cancer tissues.

The expression of PTK7 was further assessed in clinical samples from 180 patients with prostate cancer and 60 with benign prostatic hyperplasia by immunohistochemical staining. Overexpressed PTK7 was detected in 113 samples (62.78%), whereas negative or weak PTK7 immunoreactivity was observed in benign prostatic hyperplasia tissues. PTK7 protein expression was high in 5 (8.33%) of 60 patients with benign prostatic hyperplasia and 113 (62.78%) of 180 patients with prostate cancer. PTK7 protein expression was overexpressed in prostate cancer tissues compared with the benign prostatic hyperplasia tissues, and the difference was statistically significant. Representative examples of PTK7 staining are shown in Figure 2. PTK7 protein was clearly localised in the cytoplasmic compartment of the prostate cells.

#### 2.1.4. Association between PTK7 Expression and Clinicopathologic Characteristics in Prostate Cancer Patients

The correlation between PTK7 expression and clinicopathological characteristics was assessed. As shown in Table 1, the results indicated that PTK7 overexpression correlated with the lymph node metastasis (*p* = 0.023), seminal vesicle invasion (*p* = 0.002), prostate cancer stage (*p* = 0.001), the higher preoperative prostate-specific antigen (*p* < 0.001), the higher Gleason score (*p* = 0.002), angiolymphatic invasion (*p* = 0.019), and biochemical recurrence (*p* = 0.004). However, no significant association was observed between PTK7 expression and age, and surgical margin status.

**Table 1:** Clinicopathologic variables and PTK7 expression in 180 prostate cancer patients.

Variable	Group	PTK7 Expression			*p* Value
		*n*	High	Low	
Age	<70	97	60 (61.9%)	37 (38.1%)	0.782
	≥70	83	53 (63.9%)	30 (36.1%)	
Lymph Node Metastasis	Presence	17	15 (88.2%)	2 (11.8%)	0.023
	Absence	163	98 (60.1%)	65 (39.9%)	
Surgical Margin Status	Presence	14	9 (64.3%)	5 (35.7%)	0.903
	Absence	166	104 (71.7%)	62 (28.3%)	
Seminal Vesicle Invasion	Presence	35	30 (64.3%)	5 (35.7%)	0.002
	Absence	145	83 (57.2%)	62 (42.8%)	
Prostate Cancer Stage	T1	103	54 (52.4%)	49 (47.6%)	0.001
	T2/T3	77	59 (76.6%)	18 (23.4%)	
Preoperative PSA	<4	5	1 (20%)	4 (80%)	<0.001
	4–10	64	23 (35.9%)	41 (64.1%)	
	>10	111	89 (80.2%)	22 (19.8%)	
Gleason Score	<7	99	51 (51.5%)	48 (48.5%)	0.002
	7	34	24 (70.6%)	10 (29.4%)	
	>7	47	38 (80.9%)	9 (19.1%)	
Angiolymphatic Invasion	Presence	35	28 (80.0%)	7(20.0%)	0.019
	Absence	145	85 (58.6%)	60 (41.4%)	
Biochemical Recurrence	Presence	52	41 (78.8%)	11 (21.2%)	0.004
	Absence	128	72 (56.3%)	56 (43.7%)	

#### 2.1.5. Predictive Significance of PTK7 Expression in Prostate Cancer Patients with Lymph Node Metastases

To evaluate the association between elevated PTK7 expression and lymph node metastases, univariate analysis of conventional clinicopathogical variables for lymph node metastases was first performed. The presence of lymph node metastases was positively associated with preoperative prostate-specific antigen, PTK7 overexpression, and presence of angiolymphatic invasion (Table 2). A multivariate logistic regression analysis was performed to evaluate the independently predictive significance of PTK7 overexpression for lymph node metastases. The initial model included preoperative prostate-specific antigen, PTK7 overexpression, and angiolymphatic invasion which had associated with lymph node metastases, as described previously. The result showed that the presence of angiolymphatic invasion and PTK7 overexpression were independently associated with lymph node metastases (Table 3).

**Table 2:** Factors predictive of lymph node metastasis in the univariable analysis.

Variable	B	S.E.	OR	95% CI	*p* Value
Prostate Cancer Stage	0.199	0.311	1.220	0.663–2.234	0.523
Gleason Score	0.068	0.216	1.070	0.701–1.633	0.753
Preoperative Prostate-Specific Antigen	0.737	0.320	2.089	1.116–3.911	0.021
Age	0.224	0.215	1.251	0.820–1.908	0.299
Surgical Margin Status	−0.265	0.336	0.767	0.397–1.483	0.430
Presence of Seminal Vesicle Invasion	0.166	0.216	1.181	0.773–1.805	0.442
PTK7 Overexpression	0.781	0.248	2.183	1.343–3.549	0.002
Presence of Angiolymphatic Invasion	0.547	0.218	1.728	1.128–2.648	0.012

**Table 3:** Factors predictive of lymph node metastasis in the multivariable analysis.

Variable	B	S.E.	OR	95% CI	*p* Value
Preoperative Prostate-Specific Antigen	0.375	0.347	1.454	0.736–2.873	0.281
PTK7 Overexpression	0.620	0.267	1.859	1.101–3.138	0.020
Presence of Angiolymphatic Invasion	0.458	0.222	1.581	1.023–2.442	0.039

#### 2.1.6. Association between PTK7 Expression and Biochemical Recurrence-Free Survival in Patients with Prostate Cancer

**Figure 3:**
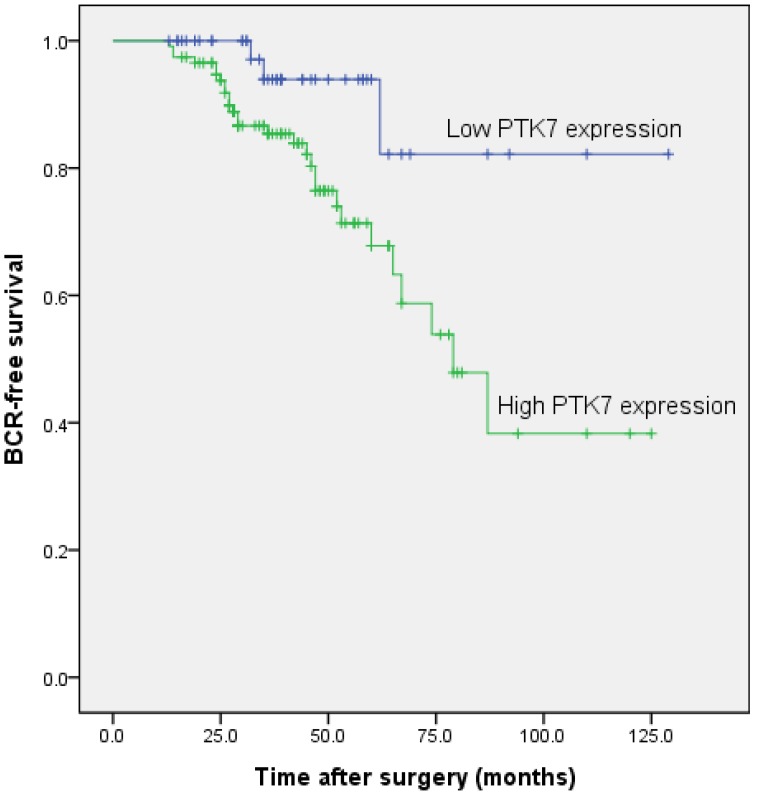
Associations between PTK7 expression and biochemical recurrence free time after radical prostatectomy in prostate cancer patients. Patients with high PTK7 expression showed significantly shorter biochemical recurrence free survival than those with low PTK7 expression (*p* < 0.001, log-rank test).

To further investigate the relation between PTK7 expression and biochemical recurrence-free survival, we compared the biochemical recurrence-free survival between PTK7 high expression and PTK7 low expression subgroups by using Kaplan-Meier analysis and log-rank test. PTK7 low expression has a survival benefit compared with high expression (Figure 3), indicating that PTK7 overexpression may be an important molecular mechanism in identifying outcomes for patients with prostate cancer. To identify the prognostic significance of clinicopathological factors for biochemical recurrence-free survival, univariate Cox analysis was conducted. PTK7 overexpression, the higher Gleason score, and seminal vesicle invasion were identified as risk factors that may affect the biochemical recurrence-free survival of patients with prostate cancer. Further adjustment of covariate factors by using multivariate Cox analysis identified PTK7 overexpression, the higher Gleason score, and seminal vesicle invasion as independent prognostic factors for biochemical recurrence-free survival. The results of the univariate and multivariate Cox analyses are shown in Table 4.

**Table 4:** Prognostic value of PTK7 expression for the biochemical recurrence-free survival in univariate and multivariate analyses by Cox regression.

Covariant	Univariate Analysis			Multivariate Analysis		
	Exp (B)	95% CI	*p* Value	Exp (B)	95% CI	*p* Value
PTK7	2.477	1.432–4.286	0.001	2.267	1.303–3.944	0.004
Gleason Score	1.703	1.280–2.265	<0.001	1.667	1.253–2.217	<0.001
Seminal Vesicle Invasion	1.505	1.132–2.003	0.005	1.371	1.029–1.827	0.031
Preoperative PSA	1.241	0.705–2.188	0.454			
Age	1.068	0.804–1.419	0.650			
Angiolymphatic Invasion	1.084	0.814–1.443	0.580			
Surgical Margin Status	1.017	0.709–1.459	0.925			
Prostate Cancer Stage	1.090	0.921–1.291	0.316			
Lymph Node Metastasis	1.140	0.850–1.528	0.381			

#### 2.1.7. Association between PTK7 Expression and Overall Survival in Patients with Prostate Cancer

Looking at the impact of PTK7 overexpression on the overall survival, we first performed univariate analysis of traditional clinicopathogical variables for prognosis. Significant variables in the overall survival analyses included PTK7 overexpression, high Gleason score, the higher preoperative prostate-specific antigen, and seminal vesicle invasion. To evaluate the independent impact of PTK7 overexpression on the overall survival, a multivariate Cox regression model adjusted for statistically significant prognostic factors was performed. Multivariate Cox regression analysis enrolling above-mentioned significant parameters showed that PTK7 protein overexpression, prostate stage, the higher Gleason score, and the higher preoperative prostate-specific antigen were independent prognostic factors for overall survival of patients with prostate cancer. The detailed results are shown in Table 5.

**Table 5:** Prognostic value of PTK7 expression for the overall survival in univariate and multivariate analyses by Cox regression.

Covariant	Univariate Analysis			Multivariate Analysis		
	Exp (B)	95% CI	*p* Value	Exp (B)	95% CI	*p* Value
PTK7	5.121	2.600–10.083	<0.001	5.650	2.843–11.229	<0.001
Gleason Score	2.526	1.788–3.568	<0.001	2.031	1.317–3.132	0.001
Preoperative PSA	2.034	1.338–23.092	0.001	1.819	1.278–2.588	0.001
Prostate Cancer Stage	4.131	2.888–5.911	<0.001	4.306	2.949–6.289	<0.001
Age	1.282	0.917–1.792	0.146			
Angiolymphatic Invasion	1.373	0.813–2.319	0.235			
Surgical Margin Status	1.101	0.703–1.724	0.674			
Lymph Node Metastasis	1.044	0.746–1.462	0.800			
Seminal Vesicle Invasion	1.358	0.956–1.928	0.087			

### 2.2. Discussion

Prostate cancer is one of the most common cancers and the second highest cause of cancer death in men in Western countries. Despite the availability of an earlier diagnosis using serum prostate-specific antigen and effective treatments, including hormone therapy, surgery, and radiation, many patients with prostate cancer subsequently die following disease progression [29]. A substantial number of prostate cancer patients demonstrate prostate-specific antigen elevation on follow-up examinations, and one-third of patients with prostate cancer develop distant metastases within several years after radical prostatectomy. Pelvic lymph node metastasis is regarded as an important prognostic factor of prostate cancer and the important predictive factor of disease recurrence after radical prostatectomy. Although there is controversy about the role of pelvic lymph node dissection for prostate cancer, an important advantage may be to determine the prognosis of patients when lymph node invasion is found and it may lead to additional therapeutic opportunities, including adjuvant hormonal therapy after radical prostatectomy [30].

The present study is the first to argue that unlike in benign prostatic hyperplasia tissues, PTK7 expression is up-regulated at both mRNA and protein levels in prostate cancer tissues. High expression level of PTK7 and its cytoplasmic localisation is significantly associated with the presence of lymph node metastases. To the best of our knowledge, an association between PTK7 overexpression and lymph node metastases has not been reported in prostate cancer. This study is the first to investigate the association between PTK7 overexpression and lymph node metastases in prostate cancers. To assess the presence of an independent association between PTK7 expression and lymph node metastases, a multivariate logistic regression analysis was performed. These results suggest that PTK7 overexpression is an important predictor of lymph node metastases.

This is the first study to investigate the impact of PTK7 overexpression on prognosis using a relatively large number of clinical samples. PTK7 expression was associated with lymph node metastases, prostate cancer stage, and Gleason score, which may prove useful in understanding disease progression of prostate cancer and the prognosis of patients with prostate cancer. Multivariate Cox regression analysis indicated that PTK7 overexpression was an independent prognostic factor for overall survival and biochemical recurrence-free survival in patients with prostate cancer. The results of this study are consistent with previously reported results. High PTK7 expression was highly associated with poor prognosis of some type of carcinomas.

In line with the possible role of PTK7 in human tumorigenesis, previous studies have proven that PTK7 overexpression was detected in several human malignant tumors. PTK7 has been identified as a protein with an important role not only in embryogenetic tube formation, but also migration and invasion of endothelial and cancer cells *in* *vitro*. Treatment with the entire extracellular domain of PTK7 acting as a decoy receptor or knockdown of PTK7 prevented vascular endothelial growth factor-induced migration, invasion, and tube formation, and angiogenesis *in* *vivo*. Knockdown of PTK7 has been shown to inhibit proliferation and invasion of liposarcoma cells and induces apoptosis. Collectively, these data suggest that PTK7 has a significant role in the oncogenesis and progression of human cancers.

## 3. Experimental Section

### 3.1. Clinical Specimens

We retrospectively reviewed the records of 180 consecutive patients of prostate cancer who underwent radical prostatectomy at the department of urology, the second hospital of Tianjin medical university, Tianjin Medical University, Tianjin, China, from January 1999 to December 2010 [30]. Informed consent was obtained from all of the patients. This study was conducted in accordance with the Declaration of Helsinki. The research ethics committee of Tianjin Medical University approved this study. All patients with prostate cancer had undergone radical prostatectomy for their disease without previous radiation therapy, neoadjuvant chemotherapy, androgen deprivation treatment, or immunotherapy. Hematoxylin and eosin stained slides that had been prepared from radical prostatectomy specimens were re-evaluated by two genitourinary pathologists who was blinded to the patients’ clinical information. The following pathological parameters were analyzed for each prostate cancer patient: Gleason score, surgical margin status, angiolymphatic invasion status, seminal vesicle invasion status, and lymph node metastasis status. The time to biochemical relapse was defined as the period between surgical treatment and the measurement of two successive values of serum prostate-specific antigen level ≥0.2 ng/mL [31]. Overall survival was defined as the period from the end of treatment to death or the time of the last follow-up [32]. In addition, fresh tissues from 40 patients, including tumor tissues (*n* = 20) and benign prostatic hyperplasia tissues (*n* = 20), were collected and stored at −80 °C immediately after resection to extract protein and RNA.

### 3.2. qRT-PCR

Briefly, RNA isolation was performed using the TRIzol reagent (Invitrogen, Carlsbad, CA, USA). cDNA was prepared using an oligo (dT) primer and reverse transcriptase following standard protocols (PrimeScript RT-PCR kit; Takara Bio, Kyoto, Japan). Human β-actin was amplified as an endogenous control. The levels of mRNA were quantified by real-time PCR with the Applied Biosystems 7900HT Fast Real-Time PCR System using SYBR Premix Ex Taq (Applied Takara Bio, Lincoln, CA, USA). The sequences of the primers were as follows: human PTK7 forward 5'-GGAAGCCACACTTCACCTAGCAG-3' and reverse 5'-CTGCCACAGTGAGCTGGACATGG-3'; human β-actin forward 5'-TGACGTGGACATCCGCAAAG-3' and reverse 5'-CTGGAAGGTGGACAGCGAGG-3'. The PCR conditions included an initial denaturation step of 94 °C for 2 min, followed by 45 cycles of 94 °C for 30 s, 57 °C for 60 s, 72 °C for 60 s. All quantitative real time reverse transcriptase polymerase chain reactions were performed in triplicate. Relative expression was presented using the 2^−ΔΔ*C*^^t^ method.

### 3.3. Western Blotting

Twenty frozen tissue sections of prostate cancer and twenty frozen tissue sample of benign prostatic hyperplasia were homogenized in lysis buffer consisting of 1% Triton X-100 and a protease inhibitor mixture in phosphate-buffered saline. The mixture was centrifuged at 12,000× *g* for 15 min at 4 °C, and the supernatant was obtained. Sixty micrograms of a protein extract was separated by 10% SDS polyacrylamide gel electrophoresis and transferred onto polyvinylidene difluoride (PVDF) filters. The filters were blocked at 4 °C overnight with blocking buffer (pH = 7.6) containing 5% nonfat dry milk and incubated with mouse anti-human monoclonal PTK7 antibody (MAB4499, R&D Systems, Minneapolis, MN, USA) overnight at 4 °C, which specifically recognizes the PTK7 protein. Immunoreactive proteins were stained using a chemiluminescence detection system. Membranes were then washed with stripping solution for 1 h and treated as described above but with β-actin antibody as an internal control.

### 3.4. Immunohistochemistry

Standard immunoperoxidase procedure was used to visualize PTK7 expression. Briefly, paraffin-embedded slides were deparaffinized in xylene and rehydrated with various concentrations of ethanol. For the antigen retrieval, slides were immersed in 0.01 M citrate buffer, pH 6, and heated in a boiling water bath for 15 min. Slides were then incubated with a mouse anti-PTK7 monoclonal antibody (MAB4499, R&D Systems, Minneapolis, MN, USA) for 1 h at room temperature. After washing with TBS, the slides were incubated with a biotinylated secondary antibody for 45 min at room temperature, rinsed in TBS and then incubated in streptavidin/horseradish peroxide for 30 min at room temperature. After rinsing in TBS, immunoreactivity was developed with 3,3'-diaminobenzidine substrate containing 0.03% hydrogen peroxide and counterstained with Harris Hematoxylin for 1 min. Negative controls were treated identically but with the primary antibody omitted.

### 3.5. Semiquantitative Analysis of PTK7 Staining

All samples were independently evaluated by two independent pathologists experienced in evaluating immunohistochemistry and who were blinded to the clinicopathologic information of these patients. PTK7 protein expression levels were classified semiquantitatively combining the proportion and intensity of positively stained immunoreactive cells. The percentage of positive-staining tumor cells was scored as follows: 0 (<5% positive tumor cells); 1 (5%–50% positive tumor cells); and 2 (>50% positive tumor cells). Staining intensity was scored as follows: 0 (no staining or only weak staining); 1 (moderate staining); and 2 (strong staining). The sum of the staining intensity score and the percentage score was used to define the PTK7 protein expression levels: 0–2, low expression and 3–4, high expression [33]. The independent scores assigned by the two independent pathologists were combined into a final score, which was reported in this study. Cases with discrepancies were re-reviewed simultaneously by the original two pathologists and a senior pathologist until a consensus was reached.

### 3.6. Statistical Analysis

All of the analyses were performed using SPSS 17.0 for Windows (SPSS, Chicago, IL, USA). For continuous variables, Student’s *t*-test was performed. The χ^2^ test was used to analyse the differences of categorical variables. Survival curves were plotted by using the Kaplan-Meier method and compared using the log-rank test. The Cox proportional hazard model was used for the multivariate analysis of the independent prognostic factors for overall survival and biochemical recurrence-free survival. Univariate and multivariate logistic regressions with covariate adjustment were used to assess the association between PTK7 overexpression and pelvic lymph node metastasis. Two-sided *p* < 0.05 was considered as statistically significant.

## 4. Conclusions

In the light of the above findings, PTK7 can, therefore, be considered as a novel biomarker of lymph node metastases and a promising therapeutic target in patients with prostate cancer. Although the results are intriguing, this investigation has limitations. The current study is a retrospective analysis with a relatively limited number of prostate cancer patients. Thus, a more thorough investigation in a larger series of patients with prostate cancer is necessary to confirm the use of this new biomarker.
